# Association of LPCAT1*rs9728 Variant with Reduced Susceptibility to Neonatal Respiratory Distress Syndrome

**DOI:** 10.3390/biomedicines13092237

**Published:** 2025-09-11

**Authors:** Shimaa Dorgham, Sohier Yahia, Doaa Shahin, Ahmad M. Eita, Eman A. Toraih, Rami M. Elshazli

**Affiliations:** 1 Department of Pediatrics, Faculty of Medicine, Mansoura University, Mansoura 35516, Egypt; shimaadorgham066@gmail.com (S.D.); sohier_yahia@yahoo.com (S.Y.); mahfo2005@hotmail.com (A.M.E.); 2 Genetics and Metabolic Unit, Mansoura University Children Hospital, Faculty of Medicine, Mansoura University, Mansoura 35516, Egypt; 3 Hematology Unit, Department of Clinical Pathology, Faculty of Medicine, Mansoura University, Mansoura 35516, Egypt; doaashahin2007@mans.edu.eg; 4 Department of Cardiovascular Perfusion, College of Health Professions, Upstate Medical University, New York, NY 13210, USA; 5 Medical Genetics Unit, Department of Histology and Cell Biology, Suez Canal University, Ismailia 41522, Egypt; 6 Department of Surgery, School of Medicine, Tulane University, New Orleans, LA 70112, USA; 7 Biochemistry and Molecular Genetics Unit, Department of Basic Sciences, Faculty of Physical Therapy, Horus University—Egypt, New Damietta 34517, Egypt; 8 Department of Biological Sciences, Faculty of Science, New Mansoura University, New Mansoura City 35742, Egypt

**Keywords:** LPCAT1*rs9728, genetic polymorphism, preterm neonates, NRDS

## Abstract

**Background/Objectives**: Neonatal respiratory distress syndrome (NRDS) is a heterogenous respiratory illness that mainly affects preterm neonates. It is characterized by insufficient production of pulmonary surfactant and impaired lung compliance. The lysophosphatidylcholine acyltransferase 1 (LPCAT1) enzyme has a crucial function in lipid remodeling through the conversion of lysophosphatidylcholine to phosphatidylcholine, the major component of pulmonary surfactant. In this research, we aimed to investigate the association of the LPCAT1*rs9728 variant with NRDS susceptibility using hereditary analysis and bioinformatic approaches. **Methods**: The *LPCAT1* (rs9728; c.*1668T>C) variant was characterized among 100 preterm neonates with RDS and 100 non-RDS neonates utilizing the TaqMan SNP genotyping assay. Logistic regression analysis was performed to identify the risk factors of respiratory distress syndrome. The functional mechanism of the *LPCAT1* gene was elucidated using bioinformatic approaches. **Results**: The LPCAT1*rs9728 C/C genotype was significantly associated with a 78% reduced risk of NRDS (OR = 0.22, *p* = 0.027), although the minor C allele did not attain a significant finding (OR = 0.83, *p* = 0.416). Apgar score and Silverman–Andersen respiratory severity score (RSS) were statistically significant with prematurity classes (*p* < 0.05). Additionally, gestational age and birth weight were considered independent risk factors in the progression of RDS among preterm neonates. **Conclusions**: This research exhibited a significant difference between the *LPCAT1* (rs9728; c.*1668T>C) variant and reduced risk against the development of RDS among preterm neonates. The rs9728*C/C genotype revealed a significant association with decreased risk of NRDS compared to non-RDS neonates.

## 1. Introduction

Neonatal respiratory distress syndrome (NRDS)—previously recognized as pulmonary hyaline membrane disease—is a life-threatening respiratory illness that predominantly affects preterm neonates (infants born alive before 37 weeks of gestation) immediately after birth [1,2]. The burden of NRDS escalates significantly with lower gestational age and birth weight, and it is a frequent contributor of neonatal mortality, especially in Africa, Asia, and the Middle East [3,4]. The clinical symptoms of NRDS were promptly recognized within the first few minutes to hours after delivery and progressively aggravated over the subsequent 72 h of life [5,6]. In Egypt, the morbidity and mortality rates among preterm neonates were elevated dramatically due to multiple considerations involving environmental, maternal, epigenetic, and hereditary issues [7,8]. The high incidence rate of NRDS is inversely correlated with the neonatal gestational age and birth weight that was attributed to impaired surfactant production enclosing the interior membranes of the pulmonary alveoli [9]. The insufficient synthesis of pulmonary surfactant from type II alveolar epithelial cells could result in elevated alveolar surface tension, disrupted gas exchange, reduced lung capacity, and poor lung compliance [10]. Pulmonary surfactant is primarily composed of combinations of phosphatidylcholine (PC), neutral lipids, and related surfactant-associated polypeptides that execute crucial mechanisms in the biological processes of surfactant metabolism and function [11]. Four surfactant-definite polypeptides were identified and designated as SP-A, SP-B, SP-C, and SP-D which are implicated in the optimization of pulmonary inflammatory reactions [12]. The synthetic mechanism of pulmonary surfactant initiates within the alveolar type II pneumocytes at the endoplasmic reticulum with phospholipids that are complexed with SP-A, SP-B, and SP-C inside the lamellar bodies and then released into the alveolar surfaces through exocytosis [9,13].

**Figure 1:**
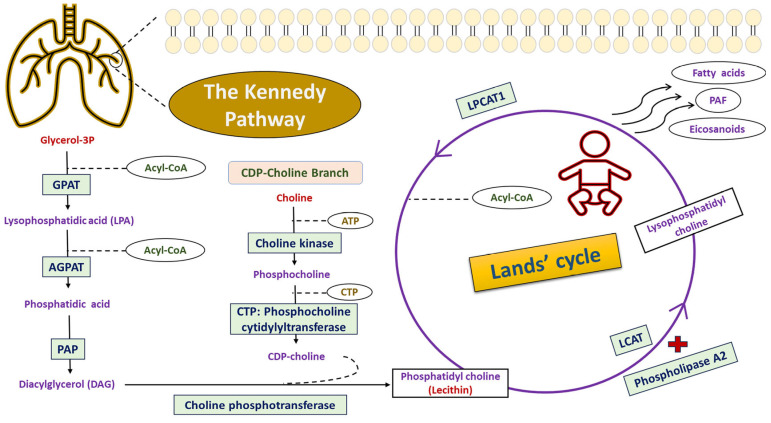
Biosynthetic pathway of phosphatidylcholine (PC) in mammalian tissues. Two metabolic processes were involved in the synthesis of phosphatidylcholine, including the de novo mechanism (Kennedy pathway) and the remodeling pathway (Lands’ cycle). In the de novo pathway, the production of lysophosphatidic acid (LPA) is achieved from the combination of glycerol-3-phosphate and acyl-CoA by the action of glycerol-3-phosphate acyltransferases (GPAT). Then, LPA is converted to phosphatidic acid using acylglycerol phosphate acyltransferases (AGPAT). The phosphatidic acid phosphatase (PAP) catalyzes the dephosphorylation reaction of phosphatidic acid, yielding diacylglycerol (DAG). The CDP-choline branch of the Kennedy pathway generates phosphatidylcholine using three enzymatic reactions. The LPCAT1 enzyme was expressed within the alveolar type II pneumocytes and executed a crucial function in the conversion of lysophosphatidylcholine to phosphatidylcholine using the remodeling pathway of Lands’ cycle. In this cyclic pathway, the lysophosphatidylcholine generated by the action of phospholipase A2 and lecithin–cholesterol acyltransferase (LCAT) was subjected to a re-acylation reaction via the incorporation of a definite fatty acid into the sn-2-position to produce new phosphatidylcholine by the action of the LPCAT1 enzyme. Abbreviations: PAF, platelet-activating factor; CTP, cytidine triphosphate; CDP, cytidine diphosphate; ATP, adenosine triphosphate, and LPCAT1, lysophosphatidylcholine acyltransferase 1.

The main constituent of pulmonary surfactant is saturated phosphatidylcholine (PC) that primarily encompasses dipalmitoyl-phosphatidylcholine (DPPC)—commonly known as lecithin. It is responsible for lowering surface tension, along with other remaining components including palmitoyl-myristoyl-phosphatidylcholine (PMPC), palmitoyl-palmitoleoyl-glycero-phosphocholine, cholesterol, phosphatidylglycerol, and surfactant polypeptides [14,15]. The metabolic biosynthesis of phosphatidylcholine is a sophisticated process that requires two fundamental processes: the de novo mechanism (Kennedy pathway) and the remodeling pathway (Lands’ cycle), as illustrated in (Figure 1) [16,17]. These metabolic processes are essential for the synthesis of multiple phospholipids, the maintenance of structural integrity of cellular membranes, and the generating of lipid precursors such as platelet-activating factors and eicosanoids [18]. Briefly, the Kennedy pathway initiates with the biosynthetic process of lysophosphatidic acid (LPA) through the condensation of glycerol-3-phosphate and acyl-CoA by the action of glycerol-3-phosphate acyltransferases (GPAT) [17,19]. LPA is then converted to phosphatidic acid using the enzymatic reaction of acylglycerol phosphate acyltransferases (AGPAT) [20,21]. This is followed by the dephosphorylation of phosphatidic acid to diacylglycerol (DAG), catalyzed by phosphatidic acid phosphatase (PAP) [22]. Additionally, the primary mechanism within this de novo mechanism involves the biosynthetic reactions of phosphatidylcholine through the CDP-choline branch [23]. The initial reaction is choline phosphorylation that is catalyzed by choline kinase yielding phosphocholine, while the subsequent enzymatic reaction is mediated by CTP: Phosphocholine cytidylyltransferase, resulting in the production of cytidine diphospho-choline (CDP-choline) [24]. Lastly, the third reaction is activated by the action of the choline phosphotransferase enzyme that catalyzes the formation of phosphatidylcholine from CDP-choline and DAG [23,24].

The lysophosphatidylcholine acyltransferase 1 (*LPCAT1*; other synonyms include AGPAT9, LPLAT8, and acyltransferase-like 2) gene is positioned on the shortened arm of chromosome #5p15.33 and spans approximately 67,843 bases (Chr.5:1,456,480–1,523,962) along the reverse strand [25,26]. This gene encoded a cytosolic enzyme with 534 residues that predominantly expressed within the alveolar type II pneumocytes and executed a crucial function in the biosynthesis of pulmonary surfactant through the modulation of lysophosphatidylcholine to phosphatidylcholine using the remodeling pathway of Lands’ cycle [27,28]. In this definite cycle, the lysophosphatidylcholine generated by the action of phospholipase A2 and lecithin–cholesterol acyltransferase (LCAT) was subjected to a re-acylation reaction via the incorporation of a certain fatty acid into the sn-2-position to produce new phosphatidylcholine by the action of the LPCAT1 enzyme [27,29]. Recently, the expression profile of the LPCAT1 enzyme has been studied in various cancerous diseases, including hepatocellular carcinoma [30], gastric carcinoma [25], endometrial cancer [31], lung adenocarcinoma [32], and breast carcinoma [33]. After precise literature screening, our team identified a single report studying the association of the *LPCAT1* (rs9728; c.*1668T>C) variant with the susceptibility to NRDS among Chinese neonates [34]. The LPCAT1*rs9728 variant was associated with NRDS under homozygous model, supporting a potential role for *LPCAT1* polymorphism with disease susceptibility [34]. Replication in Egyptian neonates is warranted due to variations in allele frequencies, genetic architecture, and linkage disequilibrium patterns across ancestries, and North African groups have been under-represented in genetic studies [35]. The newly established Egyptian genome reference highlights this population’s unique genetic background and the requirement for population-specific evidence to guide precision neonatal care [35,36]. Thus, our team was encouraged to assess this genetic variant among Egyptian neonates using hereditary analysis and bioinformatic techniques.

## 2. Materials and Methods

### 2.1. Study Design and Ethical Considerations

This hospital-based, case–control study obtained authorized permission from the Institutional Review Board of the Faculty of Medicine, Mansoura University, Mansoura, Egypt, with a recording number (MS.22.01.1834). Upon explaining the advantages and benefits of hereditary analysis in limiting the adverse effects of various respiratory illnesses, particularly neonatal respiratory distress syndrome, the parents of preterm neonates were encouraged to allocate written informed consent before enrolling in this research. Furthermore, this research fulfilled the commitments of the Helsinki Declarations.

### 2.2. Study Participants and Eligibility Criteria

This established work involved a total of 200 preterm neonates (less than 37 weeks of gestational age) categorized into two main subgroups: 100 neonates with RDS, consisting of 53.0% males and 47.0% females, and 100 unrelated non-RDS neonates without signs of respiratory distress, which included 63.0% males and 37.0% females, matched for gestational age, gender, and geographic province. Moreover, the recruitment of these preterm neonates was accomplished from the Neonatal Intensive Care Unit (NICU), Pediatric Department, Mansoura University Children’s Hospital, Mansoura, Egypt, in the period extending from October 2022 to December 2023. The diagnostic criteria of NRDS depend on a combination of various parameters, including the clinical signs of respiratory distress, chest imaging investigations, and laboratory findings [37]. The clinical manifestations of RDS in neonates include tachypnea (more than 60 breaths/minute), nasal flaring, expiratory grunting, chest retractions (subcostal, intercostal, and suprasternal), and cyanosis that necessitates supplemental oxygen [9,13,38]. Exclusion criteria for this research included aberrant genetic disorders, major congenital anomalies, inborn errors of metabolism, early-onset neonatal sepsis, intrauterine infections, and birth asphyxia [39].

### 2.3. Clinical Signs of NRDS

Preterm RDS neonates were classified according to their gestational age at delivery into four subcategories, including late preterm (34–36 weeks), moderate preterm (32–33 weeks), very preterm (28–31 weeks), and extreme preterm (<28 weeks) [40,41]. The assessment of gestational age was performed within 24 h of birth using the New Ballard score by a trained neonatologist [42]. The Apgar score was established to evaluate the general condition of neonates, the necessity for resuscitation, and neurodevelopmental inadequacies [43,44]. Additionally, the respiratory severity score (RSS) conceived by Silverman and Andersen was designed to identify the degree of severity related to respiratory distress among neonates [45].

### 2.4. Definition of Study Variables

The variables of this study were divided into two subsets involving maternal/obstetric and preterm NRDS characteristics.

#### 2.4.1. Maternal and Obstetric Characteristics

The maternal/obstetric features are classified into three subclasses, including the following subclasses: (a) sociodemographic data involving maternal age, maternal age groups, and consanguinity status; (b) obstetric factors comprising previous preterm birth, use of antenatal steroids, preterm premature rupture of membranes (PPROM), modes of delivery, and multiple pregnancy; and (c) maternal comorbidities that covered gestational diabetes mellitus (GDM), pregnancy-induced hypertension (PIH), antepartum hemorrhage, and infections. Additionally, mode of delivery included spontaneous or provider-initiated preterm birth, while infections were systemic maternal infections, bacterial vaginosis, and/or chorioamnionitis.

#### 2.4.2. Neonatal Characteristics of Preterm RDS

The characteristics of preterm NRDS were subdivided into three main subgroups, including the following subgroups: (a) demographic data including gender, gestational births, gestational age, and birth weight; (b) clinical data involving Apgar score and RSS, and use of surfactants. Furthermore, neonatal birth weights were classified into three categories, including low birth weight (≥1500 to <2500 g), very low birth weight (≥1000 to <1500 g), and extremely low birth weight (<1000 g) [46]. Lastly, the premature neonates were prospectively assessed for the following criteria: (a) respiratory outcomes including duration of mechanical ventilation, duration of oxygen therapy, and length of hospital stay; (b) neonatal comorbidities involving patent ductus arteriosus, bronchopulmonary dysplasia, retinopathy of prematurity, intraventricular hemorrhage, pulmonary hemorrhage, pneumothorax, and late-onset sepsis; and (c) survival status, either survived or dead.

### 2.5. Selection of the LPCAT1 Genetic Variant

The functional analysis and protein structure of the *LPCAT1* gene were evaluated using multiple electronic databases, including Ensembl for comparative genomic analysis (https://www.ensembl.org/), the National Center of Biotechnology Information, NCBI (https://www.ncbi.nlm.nih.gov/gene/79888/), the interactive visualization for annotated protein (https://wlab.ethz.ch/protter/), the STRING database for predicted protein interactions (https://string-db.org/), the subcellular localization database for curated annotations (https://compartments.jensenlab.org/), and the Gene Mania database for gene association networks (https://genemania.org/). Additionally, the *LPCAT1* (rs9728; c.*1668T>C) variant was annotated using VarSome (https://varsome.com/), where it is classified as benign based on American College of Medical Genetics and Genomics (ACMG) criteria. Regulatory potential was further assessed using TargetScan, miRNASNP, and PolymiRTS, which identified changes in several predicted miRNA binding sites in the variant allele. All these electronic databases were accessed on 26 April 2025.

### 2.6. Allelic Discrimination Analysis for LPCAT1*rs9728 Variant

The genomic DNA was extracted from peripheral leukocytes of blood sample for all preterm neonates utilizing the mini kit purchased form QIAamp DNA Blood (QIAGEN, Hilden, Germany, Cat. No: 51104) following the manufacturer’s guidelines. The concentration of isolated DNA was measured using the Nanodrop-1000 spectrophotometer (NanoDrop Tech., Wilmington, NC, USA) [47]. The purified DNA specimens were subjected to preservation at −80 °C until the time of genetic analysis. The genotyping of the LPCAT1*rs9728 variant was executed using TaqMan allelic discrimination analysis, as previously described [48]. The reaction assay for the LPCAT1*rs9728 variant (C___8769720_10; Applied Biosystems, Foster City, CA, USA) encompasses definite probes that could identify the (A)/(G) alleles at the chromosomal location of Chr.5:1,461,983 using the following context sequence: “[VIC/FAM] CAA CAC GCC AAG AGC CCT GAA ATT G[A/G] CTT CGG TTT ACT CCA TCC CTG TCT G” according to the assembly GRCh38. The protocol mixture was adjusted to a total volume of 25 μL containing 4 μL genomic DNA template, 1.25 μL TaqMan SNP genotyping assay Mix, 12.5 μL TaqMan Universal PCR Master Mix, and 7.25 μL DNase-free/RNase-free water. Additionally, the amplification process was accomplished within a 48-well reaction plate utilizing the StepOne™ Real-Time PCR System (Applied Biosystems, Foster City, CA, USA). The reaction conditions were adapted in four staging system involving the following stages: stage (I) incubation at 60 °C for 30 s; stage (II) polymerase activation at 95 °C for 10 min; stage (III) PCR reaction including 40 amplified cycles of denaturation at 95 °C for 15 s and annealing/extension at 60 °C for 60 s; and lastly, stage (IV) final extension step at 60 °C for 30 s. To exclude the false genotyping calls, about 10% of the original specimens were subjected to recycling within independent runs, generating concordance findings. The amplified results were interpreted using the StepOne™ software version 2.3 (Applied Biosystems, Foster City, CA, USA).

### 2.7. Statistical Analysis

The manipulation of statistical analysis in this work was performed using Stata Statistical Software, version #17 (2021, StataCorp. LLC, College Station, TX, USA), and R Studio 2023.12.1 Build 402, as previously described [49]. G*Power (version 3.1.9.7) was used to calculate the statistical power of this research, applying an α error probability of 0.05 and estimated effect sizes of 0.33 among the study participants; the calculated study power was 96% [50]. The Chi square (χ^2^), two-sample independent *t*-test, and One-Way ANOVA approaches were used to compute the parametric distribution among different established variables. The genotypic and allelic distributions of the LPCAT1*rs9728 variant were utilized to calculate the crude and adjusted odds ratio and 95% confidence intervals under various hereditary models [47]. A Bonferroni correction was applied for four genetic models tested, setting the corrected significance threshold at *p* < 0.01 (0.05/4). The selection of the best hereditary model for this potential variant was executed using Akaike information criterion (AIC) [51]. The two-tailed p-value was adjusted to be less than 0.05, resulting in significant findings. Kaplan–Meier survival curves were established to identify the survival probability among different groups [52]. Survival analyses were exploratory and based on time from birth to in-hospital death. Given the short follow-up and low event counts in some genotype groups, Kaplan–Meier curves were used for descriptive purposes only.

## 3. Results

### 3.1. Maternal and Obstetric Characteristics of the Studied Participants

The main maternal and obstetric characteristics among RDS neonates and non-RDS neonates are illustrated in Table 1. Our findings indicated that the use of antenatal steroids, modes of delivery, and multiple pregnancy revealed significant differences among RDS neonates compared to non-RDS neonates (*p* < 0.05). Additionally, pregnancy-induced hypertension and antepartum hemorrhage were associated with elevated risk of RDS compared to non-RDS neonates (*p* < 0.05). Conversely, other variables, including maternal age, consanguinity, previous preterm birth, preterm premature rupture of membranes (PPROM), gestational diabetes mellitus, and infections, did not identify significant associations among the studied participants (*p* > 0.05).

### 3.2. Preterm Neonatal Characteristics of the Studied Participants

The main demographic and clinical data among RDS neonates and non-RDS neonates are illustrated in Table 2. There was a statistically significant difference for gestational births and Apgar score among RDS neonates compared to non-RDS neonates (*p* < 0.05). The mean Silverman–Andersen respiratory severity score (RSS) in the RDS group was 7.74 ± 2.75. There were 17% with mild scores, 24% with moderate scores, and 59% with severe scores.

### 3.3. Basic Characteristics of NRDS Classes Stratified by Prematurity

The main demographic and clinical data among the NRDS groups are summarized in Table 3. There was a significant difference between lower gestational age and birth weight with increased risk of RDS among the prematurity classes (*p* < 0.05). Lower Apgar score and higher Silverman–Andersen respiratory severity score (RSS) were statistically significant with prematurity classes (*p* < 0.05).

### 3.4. RDS Preterm Neonatal Outcomes Based on Gestational Age Classes

Different neonatal outcomes including respiratory outcomes, neonatal comorbidities, or survival status among RDS neonates are depicted in Table S1. There was a statistically significant correlation between the duration of oxygen therapy, and the length of hospital stay (*p* < 0.05), with the longest duration reported for both extreme preterm and very preterm neonates. Incidence of intraventricular hemorrhage showed a statistical difference among gestational age groups (*p* < 0.05) that was higher in extreme preterm neonates. There were 25 deaths among RDS neonates, with a statistical difference among different age groups (*p* < 0.001). The highest mortality was observed in extreme preterm neonates, where 77.8% did not survive, highlighting their increased vulnerability compared to other groups.

### 3.5. Analysis of LPCAT1 (rs9728; c.*1668T>C) Variant

The genotypic and allelic distributions of the *LPCAT1* (rs9728; c.*1668T>C) variant among RDS neonates compared to non-RDS neonates are presented in Table 4. Hardy–Weinberg equilibrium (HWE) was tested in both RDS neonates and non-RDS neonates, but only control HWE was used to assess genotyping quality. This equation attained equilibrium among non-RDS neonates (*p* = 0.220), supporting reliable genotyping, whereas deviation in RDS neonates (*p* < 0.001) may reflect a genuine disease association that might be attributed due to the higher distribution of heterozygosity (T/C genotype; 71.0%).

**Figure 2:**
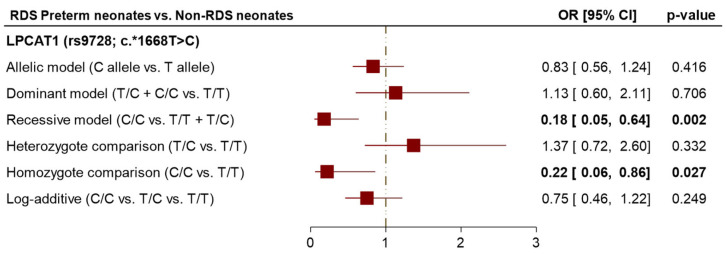
Genetic association models for *LPCAT1* (rs9728; c.*1668T>C) variant among RDS preterm neonates compared to healthy controls. Bold values indicate the *p* < 0.05.

Interestingly, the common homozygous (T/T genotype) was observed to be 29.0% among non-RDS neonates and 26.0% among RDS neonates. The more prevalent genotype is the heterozygosity (T/C genotype) that was identified in 56.0% of the non-RDS neonates compared to 71.0% of the RDS neonates (OR = 1.41, *p* = 0.332). The homozygous (C/C genotype) was observed at 15.0% among non-RDS neonates compared to 3.0% among RDS neonates (OR = 0.22, *p* = 0.027). This homozygous (rs9728*C/C genotype) was significantly associated with reduced susceptibility to the development of RDS. In terms of allelic distributions, the T allele was the more frequent, with 57.0% among non-RDS neonates, and 61.5% among RDS neonates. Conversely, the C allele was higher at 43.0% among non-RDS neonates and significantly lower at 38.5% among RDS neonates. The variant allele (C allele) did not attain considerable significance with an increased risk of RDS among the participants (OR = 0.83, *p* = 0.416) (Figure 2).

### 3.6. Impact of LPCAT1 (rs9728; c.*1668T>C) Variant with Susceptibility to NRDS

The inheritance association models for the *LPCAT1* (rs9728; c.*1668T>C) variant with the susceptibility to NRDS are illustrated in Table 4. Our findings indicated that the *LPCAT1* (rs9728; c.*1668T>C) variant provided reduced risk against NRDS development. RDS neonates carrying the double C allele within the homozygote contrast disclosed a significant association with a 78% decreased risk of NRDS (OR = 0.22, *p* = 0.027). However, after Bonferroni correction, the C/C genotype no longer reached the corrected significance threshold (*p* < 0.01), whereas the overall recessive and homozygous models remained significant. Considering the recessive model, RDS neonates carrying the rs9728*C/C genotype conferred reduced susceptibility against the development of NRDS (OR = 0.18, *p* = 0.002).

### 3.7. Implication of the LPCAT1 Variant with Demographic and Clinical Data Among RDS Neonates

The *LPCAT1* (rs9728; c.*1668T>C) variant did not achieve considerable significance with the neonatal demographic and clinical data (*p* > 0.05), as shown in Table S2.

### 3.8. Effect of the LPCAT1 Variant on Neonatal Outcomes Among RDS Neonates

The *LPCAT1* (rs9728; c.*1668T>C) variant did not achieve considerable significance with any RDS neonatal outcomes (*p* > 0.05), as shown in Table 5.

### 3.9. Survival Analysis Among the RDS Preterm Neonates

**Figure 3:**
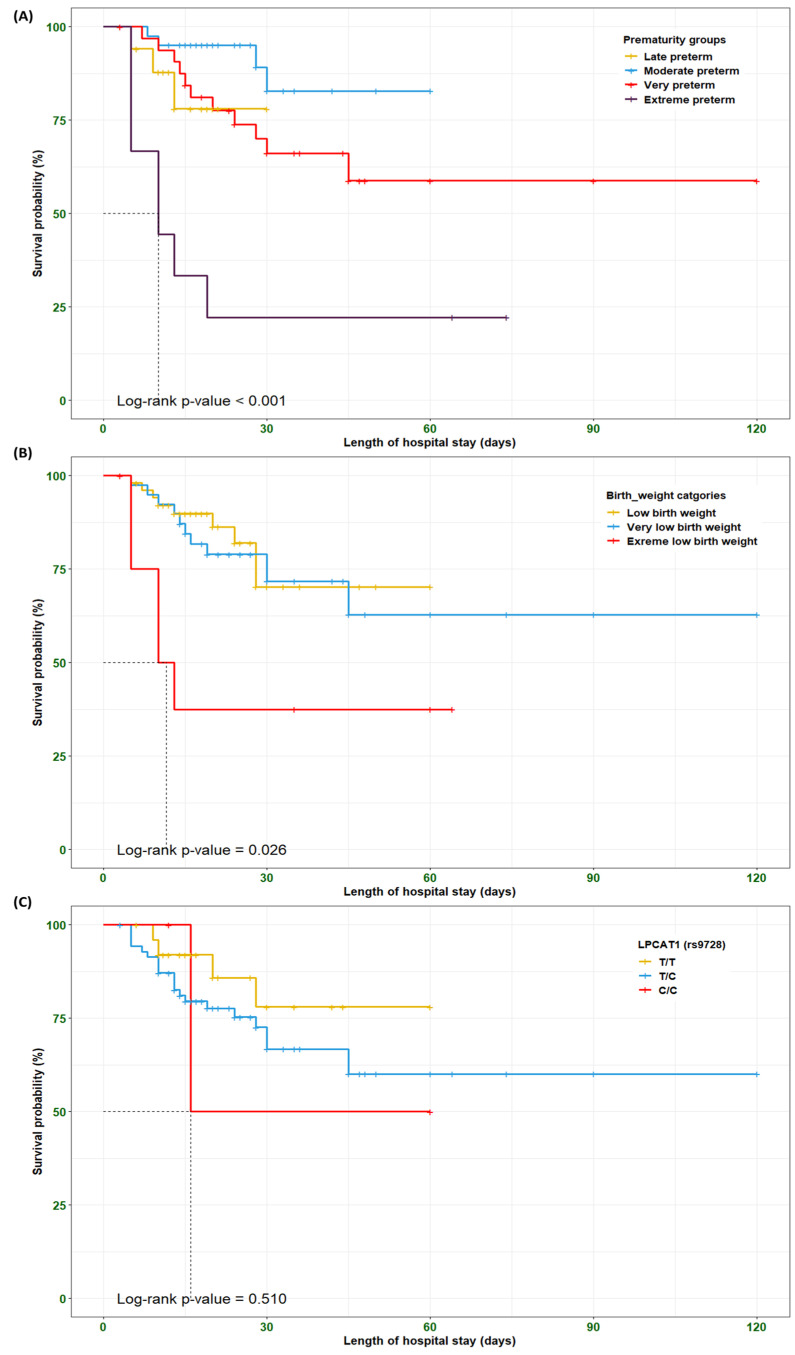
Kaplan–Meier curve of overall survival of RDS preterm neonates clustered based on (**A**) prematurity groups, (**B**) birth weight categories, and (**C**) LPCAT1*rs9726 genotypes. The log-rank *p*-value test was employed to compare the survival probability (%) of RDS preterm neonates based on the length of hospital stay.

The Kaplan–Meier curve and log-rank test were employed to identify the survival probability of RDS preterm neonates among different clustered groups, including prematurity, birth weights, and LPCAT1*rs9728 genotypes, based on the length of hospital stay. Our findings indicated that RDS preterm neonates with extreme preterm and extreme low birth weight showed shorter survival times compared to other groups (*p* < 0.001 and 0.026, respectively). Conversely, RDS preterm neonates carrying the rs9728*C/C genotype did not attain a significant difference in survival times compared to other genotypes (*p* = 0.510) (Figure 3). These findings should be viewed as exploratory given the limited number of events and the short follow-up.

### 3.10. In Silico Data Analysis for the LPCAT1 (rs9728; c.*1668T>C) Variant

**Figure 4:**
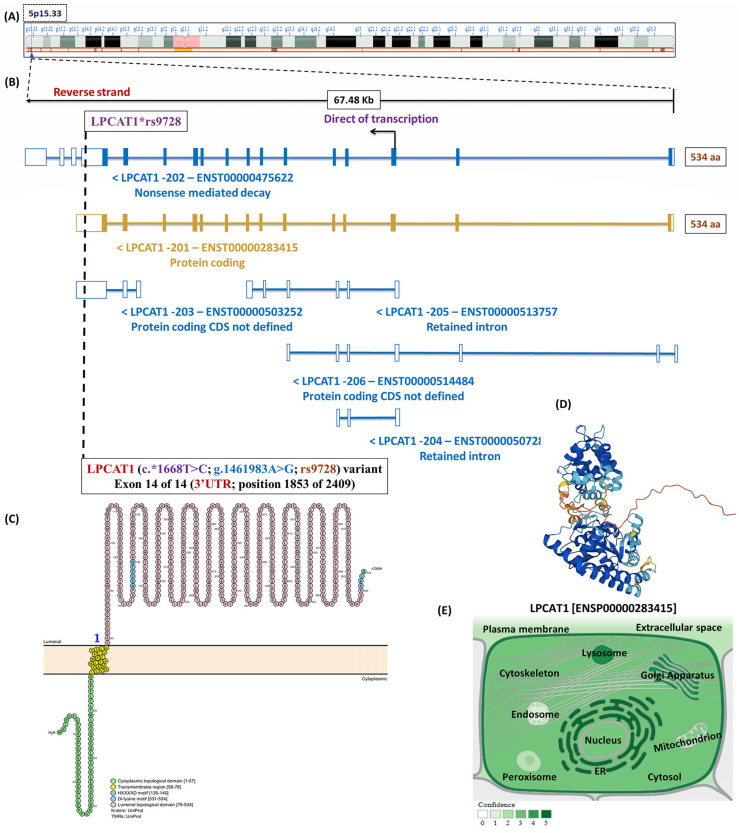

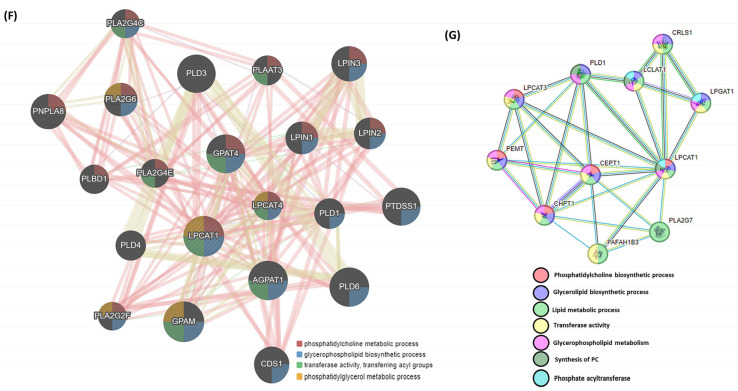
Computational bioinformatic frameworks of the *LPCAT1* gene. (**A**) The chromosomal localization of the *LPCAT1* gene. It is positioned directly on the chromosome number 5p15.33 that spanned about 67,843 bases (Chr.5:1,456,480–1,523,962) and is oriented along the reverse strand. (**B**) The genomic structure of the *LPCAT1* gene identified that it consisted of six splice transcript variants, with the main one which referred to LPCAT1-201 (ENST00000283415) and included 14 exons and 13 introns. (**C**) Amino acid residues of the LPCAT1 protein and its domains. (**D**) The predicted structure of human LPCAT1 protein designed using the AlphaFold Protein Structure database. (**E**) The subcellular localization of LPCAT1 protein. Darker color represents more abundance. (**F**) Gene–gene interaction networks of the *LPCAT1* gene. (**G**) Functional protein network analysis of the LPCAT1 protein using STRING database v12.0. [Data source: Ensembl.org, NCBI database, Protter database, Compartment database, GeneMania, and STRING database].

The computational bioinformatic frameworks for the *LPCAT1* gene and their protein interactions are displayed in Figure 4. Based on the GRCh38.p14 genome building assembly, the human *LPCAT1* gene (ENSG00000153395; other synonyms include FLJ12443, AGPAT9, AGPAT10, and LPLAT8) is situated on the shortened arm of the chromosome number 5p15.33 and transversed along the reverse strand for about 67,483 bases (Chr.5:1,456,480–1,523,962). Interestingly, the *LPCAT1* (rs9728; c.*1668T>C) variant was identified within the last exon at the 3′ untranslated region (3′ UTR). This potential gene has six splice transcripts with the independent transcript (LPCAT1-201; ENST00000283415) that included fourteen coding exons and thirteen introns (https://www.ensembl.org/). Although VarSome classifies rs9728 as benign, various bioinformatic tools suggest that the variant may alter miRNA binding motifs and potentially affect LPCAT1 transcript stability. The coding regions in this gene encoded about 534 amino acid residues (https://wlab.ethz.ch/protter/). The subcellular localization database for annotated proteins confirmed the availability of the LPCAT1 enzyme in the endoplasmic reticulum, lysosomes, and Golgi apparatus with considerable abundance (https://compartments.jensenlab.org/). The predicted genomic annotation was evaluated using the GeneMANIA database to identify the functional analysis and genetic localization of the *LPCAT1* gene with multiple metabolic reactions involving phosphatidylcholine metabolic processes, glycerophospholipid biosynthetic processes, transferring acyl groups, and phosphatidylglycerol metabolic processes (https://genemania.org/). Finally, the analysis of protein–protein interactions demonstrated how this crucial enzyme contributes to phosphatidylcholine biosynthesis, lipid biosynthetic processes, transferase activity, glycerophospholipid metabolism, and phosphate acyltransferase (https://string-db.org/). All these electronic databases were accessed on 26 April 2025.

## 4. Discussion

The etiology and pathophysiology of respiratory distress syndrome among preterm neonates arise from multiple metabolic and structural considerations that are attributed to the insufficient production of pulmonary surfactant leading to serious pulmonary dysfunctions [53,54]. The enzymatic mechanism of LPCAT1 is crucial for the biosynthesis of pulmonary surfactant by type II pneumocytes, guaranteeing the optimum functioning of the lungs within preterm neonates [55]. Following a comprehensive literature search for articles investigating the contribution of the *LPCAT1* (rs9728; c.*1668T>C) variant to the susceptibility of NRDS among different ethnic populations, this retrospective research was the outstanding study that assessed the association of this noncoding variant within the 3′UTR with the susceptibility and severity of respiratory distress syndrome among Egyptian preterm neonates. The primary findings of our work revealed the correlation of the *LPCAT1* rs9728 variant with a decreased risk of NRDS among Egyptian preterm neonates. Interestingly, this noncoding variant provided decreased susceptibility against respiratory distress syndrome in preterm neonates when compared to the non-RDS group, as shown by the homozygote comparison (OR = 0.22, *p* = 0.006) and the recessive model (OR = 0.18, *p* = 0.002). However, the variant C allele failed to attain statistical association with a decreased risk of NRDS (*p* > 0.05). Additionally, the *LPCAT1* (rs9728; c.*1668T>C) variant did not observe a notable association with the demographic data, clinical data, and neonatal outcomes among the RDS preterm neonates. In accordance with our results, we identified a single report that studied the association of the *LPCAT1* (rs9728; c.*1668T>C) variant with the risk of respiratory distress syndrome among Chinese preterm neonates [34]. In this previous study, they confirmed the correlation of the *LPCAT1* rs9728 variant with decreased risk of NRDS under the homozygote comparison (C/C vs. T/T: OR = 0.38, *p* = 0.002) and the allelic model (C allele vs. T allele: OR = 0.67, *p* = 0.003). Moreover, they observed a statistically significant association between the *LPCAT1* rs9728 polymorphism and intraventricular hemorrhage (IVH) among Chinese preterm neonates (*p* = 0.039) [34].

Furthermore, the levels of *LPCAT1* mRNA were significantly lower among newborn mice with *LPCAT1* TC/TC and associated with enzymatic activity, saturated phosphatidylcholine, and disease survival [56]. The LPCAT1 enzyme particularly interacts with specific phospholipid transfer protein (StarD10) to facilitate the trafficking process of DPPC from the endoplasmic reticulum to the lamellar bodies for storage and further secretion [57]. While rs9728 is benign under ACMG classification, its presence in the 3′ UTR may influence LPCAT1 expression through altered miRNA binding or mRNA stability—mechanisms supported by miRNA target predictions [58].

The amphipathic nature of dipalmitoyl-phosphatidylcholine as a pulmonary surfactant is attributed to its saturated side chains, which could generate highly organized and tightly packed films for persistent intervals, resulting in lowering surface tension within the alveolar lungs [59]. In this research, our findings revealed that the use of antenatal steroids, vaginal delivery, multiple pregnancies, pregnancy-induced hypertension, and antepartum hemorrhage were significantly associated with RDS neonates compared to non-RDS neonates. These results align with previous reports and confirm the involvement of RDS preterm neonates with neonatal sepsis, vaginal delivery, and antepartum hemorrhage [60,61]. Our team disclosed a significant association between lower gestational age and birth weight classes with increased risk of RDS among the prematurity categories. The Apgar score and Silverman–Andersen respiratory severity score (RSS) were statistically associated with prematurity classes. Additionally, we encountered a study conducted among Chinese preterm neonates with RDS that revealed a statistically significant correlation with gestational age, birth weight, and Apgar score compared to non-RDS neonates (*p* < 0.05) [62]. Moreover, gestational age, birth weight in grams, vaginal delivery, and premature rupture of membrane were statistically significant among Afghani preterm neonates with RDS compared to neonates without RDS (*p* < 0.05) [60].

The *LPCAT1* mRNA copies were correlated with the counts of amniotic fluid lamellar bodies [63,64]. The overexpression of *LPCAT1* mRNA was associated with multiple disorders, including hepatocellular carcinoma [30], endometrial cancer [31], pulmonary emphysema [65], lung carcinoma [66], epithelioid trophoblastic tumors [67], and breast carcinoma [26]. From the above-mentioned findings, we might speculate that the *LPCAT1* (rs9728; c.*1668T>C) variant was correlated with reduced susceptibility against the development of respiratory distress syndrome among preterm neonates. To better understand these discrepancies, additional constructed reports are required to shed light on the functional mechanism of the LPCAT1 enzyme in the progression of respiratory distress syndrome and lipid remodeling among premature neonates. The main advantages of this study include the employing of case–control design with plenty of clinical variables, utilization of multiple hereditary association models, along with combination with bioinformatic approaches to validate these outcomes. On the other hand, minor limitations were raised and addressed in the form of the small number of homozygous minor-allele (C/C) genotypes among RDS neonates, reflects underlying allele frequencies, genetic architecture, and linkage disequilibrium patterns in the Egyptian population. While statistical power calculations using G*Power confirmed sufficient power for the observed effect size, the precision of the recessive estimates is limited. Importantly, while the recessive model retained significance after correction, the association for the C/C genotype did not, and thus reflected both the small subgroup size and the conservative multiple testing adjustment. Our Kaplan–Meier survival analysis was limited by the short duration of follow-up (length of hospital stay) and the low number of deaths in some genotypic groups; therefore, these results should be considered descriptive rather than confirmatory. Another limitation is the lack of data on parental genetic history, environmental exposures (e.g., smoking, air pollution), and maternal nutritional status, which could act as unmeasured confounders of NRDS risk. Although bioinformatic analyses suggested potential regulatory roles for rs9728 in the 3′UTR of *LPCAT1* gene, we did not perform functional validation experiments (e.g., qPCR of neonatal lung tissue, protein quantification, or luciferase reporter assays). Such experiments would be valuable in confirming whether this variant directly alters *LPCAT1* expression or miRNA binding, thereby influencing NRDS risk. Therefore, replication in larger, multi-ethnic cohorts, and functional validation are warranted to more comprehensively evaluate these genetic associations.

## 5. Conclusions

This research demonstrated a genetic association between the *LPCAT1* (rs9728; c.*1668T>C) variant and respiratory distress syndrome among Egyptian preterm neonates. The *LPCAT1* C/C genotype was positively associated with reduced susceptibility to NRDS, while no significant associations were observed between rs9728 genotypes and clinical outcomes among NRDS neonates. Validation in larger, multi-center, and ethnically diverse cohorts will be essential to confirm these findings and to clarify the role of this variant in NRDS susceptibility.

## Figures and Tables

**Table 1:** Maternal and obstetric characteristics among RDS neonates compared to non-RDS neonates.

Characteristics	Levels	Study Participants		*p*
		Non-RDS Neonates	RDS Neonates	
		(n = 100)	(n = 100)	
**Demographic and clinical characteristics**				
Maternal age (years)	M ± SD	26.1 ± 5.26	27.6 ± 5.85	0.058
Maternal age groups	≤20 years, n (%)	13 (13.0)	9 (9.0)	0.090
	21–34 years, n (%)	79 (79.0)	73 (73.0)	
	≥35 years, n (%)	8 (8.0)	18 (18.0)	
Consanguinity (yes/no)	n (%)/n (%)	20 (20.0)/80 (80.0)	22 (22.0)/78 (78.0)	0.728
**Obstetric factors**				
Previous preterm birth (yes/no)	n (%)/n (%)	18 (18.0)/82 (82.0)	14 (14.0)/86 (86.0)	0.440
Use of antenatal steroids (yes/no)	n (%)/n (%)	90 (90.0)/10 (10.0)	75 (75.0)/25 (25.0)	**0.005**
PPROM (yes/no)	n (%)/n (%)	25 (25.0)/75 (75.0)	36 (36.0)/64 (64.0)	0.091
Modes of delivery (CS/VD)	n (%)/n (%)	91 (91.0)/9 (9.0)	78 (78.0)/22 (22.0)	**0.011**
Multiple pregnancy (yes/no)	n (%)/n (%)	2 (2.0)/98 (98.0)	44 (44.0)/56 (56.0)	**<0.001**
**Maternal comorbidities**				
GDM (yes/no)	n (%)/n (%)	8 (8.0)/92 (92.0)	7 (7.0)/93 (93.0)	0.788
PIH (yes/no)	n (%)/n (%)	12 (12.0)/88 (88.0)	32 (32.0)/68 (68.0)	**<0.001**
Antepartum hemorrhage (yes/no)	n (%)/n (%)	3 (3.0)/97 (97.0)	13 (13.0)/87 (87.0)	**0.009**
Infections (yes/no)	n (%)/n (%)	2 (2.0)/98 (98.0)	6 (6.0)/94 (94.0)	0.149

Data are expressed as numbers (%) or mean (SD). Chi square (χ^2^) test and two-sample independent *t*-test were applied. Abbreviations: RDS, respiratory distress syndrome; M: mean; SD, standard deviation; PPROM, preterm premature rupture of membranes; CS, cesarean section; VD, vaginal delivery; GDM, gestational diabetes mellitus; PIH, pregnancy-induced hypertension. Modes of delivery (CS/VD) included spontaneous or providers-initiated preterm birth; infections included systemic maternal infections, bacterial vaginosis, and chorioamnionitis. Bold values indicate the *p* < 0.05.

**Table 2:** Preterm neonatal characteristics among RDS neonates compared to non-RDS neonates.

Characteristics	Levels	Study Participants		*p*
		Non-RDS Neonates	RDS Neonates	
		(n = 100)	(n = 100)	
**Demographic data**				
Gender (males/females)	n (%)/n (%)	63 (63.0)/37 (37.0)	53 (53.0)/47 (47.0)	0.152
Gestational births (Singleton/Multiple)	n (%)/n (%)	98 (98.0)/2 (2.0)	56 (56.0)/44 (44.0)	**<0.001**
Gestational age (weeks)	M ± SD	32.4 ± 1.62	31.2 ± 2.54	0.452
Birth weight (grams)	M ± SD	1564.2 ± 152.3	1491.5 ± 419.3	0.645
**Clinical data**				
Apgar score	Apgar score (1 min), M ± SD	8.02 ± 0.87	6.54 ± 1.07	**<0.001**
	Apgar score (5 min), M ± SD	9.99 ± 0.10	8.91 ± 1.44	**<0.001**
Silverman–Andersen respiratory severity score (RSS)				
RSS	M ± SD	--	7.74 ± 2.75	NA
RD grades based on RSS	None or mild score (0–3), n (%)	--	17 (17.0)	NA
	Moderate score (4–6), n (%)	--	24 (24.0)	
	Severe score (7–10), n (%)	--	59 (59.0)	
Use of surfactant (yes/no)	n (%)/n (%)	--	16 (16.0)/84 (84.0)	NA

Data are expressed as numbers (%) or mean (SD). Chi square (χ^2^) test and two-sample independent *t*-test were applied. Abbreviations: RDS, respiratory distress syndrome; RD, respiratory distress; M, mean; SD, standard deviation; NA, not applicable. Bold values indicate the *p* < 0.05.

**Table 3:** Characteristics of RDS neonates based on gestational age classes.

Characteristics	Levels	Prematurity Based on Gestational Age				*p*
		Late Preterm (34–36 Weeks)	Moderate Preterm (32–33 Weeks)	Very Preterm (28–31 Weeks)	Extreme Preterm (<28 Weeks)	
		(n = 17)	(n = 41)	(n = 33)	(n = 9)	
**Demographic data**						
Gender (males/females)	n (%)/n (%)	8 (47.1)/9 (52.9)	26 (63.4)/15 (36.6)	17 (51.5)/16 (48.5)	2 (22.2)/7 (77.8)	0.140
Gestation births (Singleton/Multiple)	n (%)/n (%)	7 (41.2)/10 (58.8)	19 (46.3)/22 (53.7)	14 (42.4)/19 (57.6)	4 (44.4)/5 (55.6)	0.981
Gestational age (weeks)	M ± SD	34.4 ± 0.70	32.5 ± 0.51	29.4 ± 0.94	26.0 ± 0.87	**<0.001**
Birth weight (grams)	M ± SD	1954.4 ± 400.7	1571.5 ± 298.8	1300.7 ± 296.6	951.7 ± 299.4	**<0.001**
Birth weight classes						
LBW (≥1500–<2500)	n (%)	14 (82.4)	26 (63.4)	11 (33.3)	1 (11.1)	**<0.001**
VLBW (≥1000–<1500)	n (%)	3 (17.6)	15 (36.6)	18 (54.6)	3 (33.3)	
ELBW (<1000)	n (%)	0 (0.0)	0 (0.0)	4 (12.1)	5 (55.6)	
**Clinical data**						
Apgar score	Apgar score (1 min.), M ± SD	6.76 ± 0.66	6.75 ± 0.77	6.39 ± 1.32	5.67 ± 1.41	**0.026**
	Apgar score (5 min.), M ± SD	9.65 ± 0.99	9.29 ± 1.27	8.51 ± 1.46	7.22 ± 1.20	**<0.001**
Silverman–Andersen respiratory severity score (RSS)						
RSS	M ± SD	5.41 ± 2.72	6.97 ± 2.81	9.27 ± 1.57	10.0 ± 0.01	**<0.001**
RD grades based on RSS	None or mild score (0–3), n (%)	8 (47.1)	9 (22.0)	0 (0.0)	0 (0.0)	**<0.001**
	Moderate score (4–6), n (%)	5 (29.4)	13 (31.7)	6 (18.2)	0 (0.0)	
	Severe score (7–10), n (%)	4 (23.5)	19 (46.3)	27 (81.8)	9 (100.0)	
Use of surfactant (yes/no)	n (%)/n (%)	2 (11.8)/15 (88.2)	6 (14.6)/35 (85.4)	7 (21.2)/26 (78.8)	1 (11.1)/8 (88.9)	0.774

Data are expressed as numbers (%) or mean (SD). Chi square (χ^2^) and One-Way ANOVA tests were performed. Abbreviations: RDS, respiratory distress syndrome; LBW, low birth weight; VLBW, very low birth weight; ELBW, extremely low birth weight; RD, respiratory distress. Bold values indicate the *p* < 0.05.

**Table 4:** The genotypic/allelic frequencies of the *LPCAT1* (rs9728; c.*1668T>C) variant among the study participants.

*LPCAT1*-rs9728 Variant		Non-RDS Neonates	RDS Neonates	OR (95% CI)	*p*	
**Genotypic Frequencies**		**n (%) = 100**	**n (%) 100**			
T/T		29 (29.0)	26 (26.0)	1.0		
T/C		56 (56.0)	71 (71.0)	1.41 (0.75–2.67)	0.332	
C/C		15 (15.0)	3 (3.0)	**0.22 (0.06–0.86)**	**0.027**	
HWE		χ^2^ = 2.03, *p* = 0.220	χ^2^ = 24.9, *p* < **0.001**			
**Allelic Frequencies**		**n (%) 200**	**n (%) 200**			
T allele		114 (57.0)	123 (61.5)	1.0		
C allele		86 (43.0)	77 (38.5)	0.83 (0.56–1.24)	0.416	
**Genetic Association Models**		**Non-RDS Neonates**	**RDS Neonates**	**Adjusted OR (95% CI)**	***p***	**AIC**
**Model**	**Genotypes**	**n (%) 100**	**n (%) 100**			
Codominant	T/T	29 (29.0)	26 (26.0)	1.0	**0.006**	273.1
	T/C	56 (56.0)	71 (71.0)	1.37 (0.72–2.60)		
	C/C	15 (15.0)	3 (3.0)	**0.22 (0.06–0.86)**		
Dominant	T/T	29 (29.0)	26 (26.0)	1.0	0.706	281.1
	T/C + C/C	71 (71.0)	74 (74.0)	1.13 (0.60–2.11)		
Recessive	T/T + T/C	85 (85.0)	97 (97.0)	1.0	**0.002**	272.0
	C/C	15 (15.0)	3 (3.0)	**0.18 (0.05–0.64)**		
Log-additive	--	--	--	0.75 (0.46–1.22)	0.249	279.9

Data is expressed as numbers with percentages. Fisher’s exact and Chi square tests were applied. Abbreviations: RDS, respiratory distress syndrome; HWE, Hardy–Weinberg equilibrium; OR, odds ratio; CI, confidence intervals; AIC, Akaike information criterion. A Bonferroni correction was applied for the four genetic models tested (codominant, dominant, recessive, and log-additive), setting the corrected significance threshold at *p* < 0.01 (0.05/4). After correction, the recessive model remained significant, whereas the C/C genotype alone did not reach corrected significance. Bold values indicate the *p* < 0.05.

**Table 5:** Effect of genotypic frequencies of *LPCAT1* (rs9728; c.*1668T>C) variant on neonatal outcomes among RDS neonates.

Parameter	Levels	*LPCAT1* (rs9728; c.*1668T>C)			*p*
		T/T (n = 26)	T/C (n = 71)	C/C (n = 3)	
**Respiratory outcomes**					
Duration of MV (Days)	M ± SD	8.06 ± 4.44	9.31 ± 4.71	7.00 ± 0.01	0.568
Duration of oxygen therapy (Days)	M ± SD	14.1 ± 8.47	14.8 ± 8.62	11.3 ± 4.62	0.762
LOS (Days)	M ± SD	25.2 ± 15.0	26.2 ± 19.6	29.3 ± 26.6	0.928
**Neonatal comorbidities**					
PDA (yes/no)	n (%)/n (%)	2 (7.7)/24 (92.3)	4 (5.6)/67 (94.4)	0 (0.0)/3 (100.0)	0.843
BPD (yes/no)	n (%)/n (%)	0 (0.0)/26 (100.0)	2 (2.8)/69 (97.2)	0 (0.0)/3 (100.0)	0.659
ROP (yes/no)	n (%)/n (%)	7 (26.9)/19 (73.1)	17 (23.9)/54 (76.1)	0 (0.0)/3 (100.0)	0.586
IVH (yes/no)	n (%)/n (%)	1 (3.9)/25 (96.1)	6 (8.4)/65 (91.6)	0 (0.0)/3 (100.0)	0.653
Pulmonary hemorrhage (yes/no)	n (%)/n (%)	0 (0.0)/26 (100.0)	4 (5.6)/67 (94.4)	0 (0.0)/3 (100.0)	0.427
Pneumothorax (yes/no)	n (%)/n (%)	2 (7.7)/24 (92.3)	7 (9.9)/64 (90.1)	0 (0.0)/3 (100.0)	0.813
Late-onset sepsis (yes/no)	n (%)/n (%)	12 (46.2)/14 (53.8)	45 (63.4)/26 (36.6)	2 (66.7)/1 (33.3)	0.301
**Survival status**					
Survived	n (%)	22 (84.6)	51 (71.8)	2 (66.7)	0.412
Dead	n (%)	4 (15.4)	20 (28.2)	1 (33.3)	

Data are expressed as numbers (%) or mean (SD). Chi square (χ^2^) and One-Way ANOVA tests were performed. Abbreviations: RDS, respiratory distress syndrome; LOS, length of hospital stay; MV; mechanical ventilation; PDA, patent ductus arteriosus; BPD, bronchopulmonary dysplasia; ROP, retinopathy of prematurity; IVH, intraventricular hemorrhage. Length of hospital stay (LOS) is the time between hospital admission and discharge. Seventy-six RDS neonates were mechanically ventilated. Preterm neonates that died in the hospital and death summary was written on a report were considered as dead, while those who remained alive after 28 days of neonatal period and/or those discharged with improvement were considered as survived. Late-onset sepsis (LOS) is defined as the occurrence of sepsis beyond three days after birth.

## Data Availability

Data are available from the corresponding author upon reasonable request.

## Supplementary Materials

The following supporting information can be downloaded at https://www.mdpi.com/article/10.3390/biomedicines13092237/s1, Table S1: RDS neonatal outcomes based on gestational age classes, Table S2: Genotypic frequencies of *LPCAT1* (rs9728; c.*1668T>C) variant stratified by the demographic and clinical data of RDS preterm neonates.

## Abbreviations

The following abbreviations are used in this manuscript:

LPCAT1	Lysophosphatidylcholine acyltransferase 1
NRDS	Neonatal Respiratory Distress Syndrome
LCAT	Lecithin–cholesterol acyltransferase
DPPC	Dipalmitoyl-phosphatidylcholine
