# Supplementary material for: Association of LPCAT1*rs9728 Variant with Reduced Susceptibility to Neonatal Respiratory Distress Syndrome
Source: Biomedicines. 2025 Sep 11;13(9):2237. doi: 10.3390/biomedicines13092237 (PMC12467284; doi:10.3390/biomedicines13092237)
Supplement: Supplementary file 1 [file biomedicines-13-02237-s001.zip › biomedicines-3781326-supplementary.pdf]

Supplementary Tables

Table S1. RDS neonatal outcomes based on gestational age classes.

Characteristics	Levels	Prematurity Based on Gestational Age				*p*
		Late Preterm (34–36 Weeks) (n = 17)	Moderate Preterm (32–33 Weeks) (n = 41)	Very Preterm (28–31 weeks) (n = 33)	Extreme Preterm (<28 Weeks) (n = 9)	
**Respiratory outcomes**						
Duration of MV (days)	M ± SD	6.28 ± 2.21	8.50 ± 4.73	9.73 ± 4.79	10.3 ± 4.53	0.236
Duration of oxygen therapy (days)	M ± SD	8.12 ± 4.95	13.6 ± 6.25	18.9 ± 9.59	14.1 ± 10.1	**<0.001**
Length of hospital stay (days)	M ± SD	15.0 ± 7.27	24.5 ± 10.4	34.4 ± 24.1	22.8 ± 26.7	**0.003**
**Clinical data**						
PDA (yes/no)	M ± SD	1 (5.9)/16 (94.1)	2 (4.9)/39 (95.1)	3 (9.1)/30 (90.9)	0 (0.0)/9 (100.0)	0.747
BPD (yes/no)	M ± SD	0 (0.0)/17 (100.0)	1 (2.4)/40 (97.6)	1 (3.0)/32 (97.0)	0 (0.0)/9 (100.0)	0.861
ROP (yes/no)	M ± SD	2 (11.8)/15 (88.2)	7 (17.1)/34 (82.9)	13 (39.4)/2(60.6)	2 (22.2)/7 (77.8)	0.079
IVH (yes/no)	n (%)	0 (0.0)/17 (100.0)	2 (4.9)/39 (95.1)	1 (3.0)/32 (97.0)	4 (44.4)/5 (55.6)	**<0.001**
Pulmonary hemorrhage (yes/no)	n (%)	1 (5.9)/16 (94.1)	0 (0.0)/41 (100.0)	3 (9.1)/30 (90.9)	0 (0.0)/9 (100.0)	0.215
Pneumothorax (yes/no)	n (%)	0 (0.0)/17 (100.0)	3 (7.3)/38 (92.7)	4 (12.1)/29(87.9)	2 (22.2)/7 (77.8)	0.247
Late-onset sepsis (yes/no)	n (%)/n (%)	8 (47.1)/9 (52.9)	20 (48.8)/21 (51.2)	23 (69.7)/1(30.3)	8 (88.9)/1 (11.1)	0.054
**Survival status**						
Survived	n (%)	14 (82.4)	37 (90.2)	22 (66.7)	2 (22.2)	**<0.001**
Dead	n (%)	3 (17.6)	4 (9.8)	11 (33.3)	7 (77.8)	

Data are expressed as numbers (%) or mean (SD). Chi square (χ^2) and One-Way ANOVA tests were performed. **Abbreviations**: RDS, respiratory distress syndrome; LBW, low birth weight; VLBW, very low birth weight; ELBW, extremely low birth weight; MV; mechanical ventilation; PDA, patent ductus arteriosus; BPD, bronchopulmonary dysplasia; ROP, retinopathy of prematurity; IVH, intraventricular hemorrhage. Length of hospital stay (LOS) is the time between hospital admission and discharge. Seventy-six RDS neonates were mechanically ventilated. Preterm neonates that died in the hospital and death summary was written on a report were considered as dead, while those who remained alive after 28 days of neonatal period and/or those discharged with improvement were considered as survived. Late-onset sepsis (LOS) is defined as the occurrence of sepsis beyond three days after birth. Bold values indicate the $p < 0.05$.

Table S2. Genotypic frequencies of *LPCAT1* (rs9728; c.*1668T>C) variant stratified by the demographic and clinical data of RDS preterm neonates.

Parameter	Levels	*LPCAT1 (rs9728; c.*1668T>C)*			*p*
		T/T (n = 26)	T/C (n = 71)	C/C (n = 3)	
**Demographic data**					
Gender (Males/Females)	n (%)/n (%)	15 (57.7)/11 (42.3)	36 (50.7)/35 (49.3)	2 (66.7)/1 (33.3)	0.739
Gestation births (Singleton/Multiple)	n (%)/n (%)	13 (50.0)/13 (50.0)	31 (43.7)/40 (56.3)	0 (0.0)/3 (100.0)	0.254
Gestational age (weeks)	M ± SD	31.8 ± 2.25	30.9 ± 2.65	32.0 ± 1.73	0.353
**Prematurity categories**					
Late preterm (34–36 weeks)	n (%)	7 (26.9)	10 (14.1)	0 (0.0)	0.577
Moderate preterm (32–33 weeks)	n (%)	11 (42.3)	28 (39.4)	2 (66.7)	
Very preterm (28–31 weeks)	n (%)	7 (26.9)	25 (35.2)	1 (33.3)	
Extreme preterm (<28 weeks)	n (%)	1 (3.9)	8 (11.3)	0 (0.0)	
**Birth weight (grams)**	M ± SD	1598.1 ± 416.9	1449.9 ± 420.6	1550.0 ± 350.0	0.298
**Birth weight classes**					
LBW (≥1500–<2500)	n (%)	16 (61.5)	34 (47.9)	2 (66.7)	0.650
VLBW (≥1000–<1500)	n (%)	9 (34.6)	29 (40.9)	1 (33.3)	
ELBW (<1000)	n (%)	1 (3.9)	8 (11.3)	0 (0.0)	
**Clinical data**					
Apgar score (1 min.)	M ± SD	6.85 ± 0.73	6.44 ± 1.15	6.33 ± 1.15	0.234
Apgar score (5 min.)	M ± SD	9.12 ± 1.31	8.83 ± 1.49	9.00 ± 1.73	0.691
RSS	M ± SD	7.07 ± 2.80	8.08 ± 2.63	5.33 ± 4.04	0.085
**RD grades based on RSS**					
None or mild score (0–3)	n (%)	6 (23.1)	9 (12.7)	2 (66.7)	0.115
Moderate score (4–6)	n (%)	7 (26.9)	17 (23.9)	0 (0.0)	
Severe score (7–10)	n (%)	13 (50.0)	45 (63.4)	1 (33.3)	
Use of surfactant (yes/no)	n (%)/n (%)	6 (23.1)/20 (76.9)	9 (12.7)/62 (87.3)	1 (33.3)/2 (66.7)	0.329

Data are expressed as numbers (%) or mean (SD). Chi square (χ^2) and One-Way ANOVA tests were performed. **Abbreviations:** RDS, respiratory distress syndrome; LBW, low birth weight; VLBW, very low birth weight; ELBW, extremely low birth weight; RD, respiratory distress.
